# Applications of the MVWG Multivariable Stochastic Weather Generator

**DOI:** 10.1155/2013/571367

**Published:** 2013-07-25

**Authors:** Nándor Fodor, Ildikó Dobi, János Mika, László Szeidl

**Affiliations:** ^1^Agricultural Institute, Centre for Agricultural Research, HAS, Brunszvik street 2, Martonvásár 2462, Hungary; ^2^Hungarian Meteorological Service, Kitaibel Pál street 1, Budapest 1024, Hungary; ^3^Eszterházy Károly College, Department of Geography, Eszterházy square 1, Eger 3300, Hungary; ^4^Óbuda University, 96/B Bécsi street, Budapest 1034, Hungary

## Abstract

Weather generators (WG) became significant modules of crop models and decision support systems in the past decade. Using a large meteorological database from North America; two basic problems, related to the applicability of WGs in case of short or lacking data series, were investigated in the framework of the Multivariable weather generator (MVWG). First, the minimum data series length, required for adequate parameterization of the WG, was determined. Our results suggest that 15 years of observed data are enough for adequate parameterization of the MVWG. We then investigated a possibility of spatial interpolation of WG parameters using the outputs of the WG for sites with no meteorological observations. Coupled with the presented interpolation technique, MVWG was able to generate realistic weather data for sites with no measurements situated in climatically and geographically homogeneous regions.

## 1. Introduction

Artificial weather data series produced by stochastic weather generators can be used in a wide range of agro-meteorological, hydrological, risk analysis, and climate change studies. Combined with spatial interpolation methods, generators can provide realistic weather data for locations with no measurements using the data of the surrounding meteorological stations. Coupled with future climate scenarios weather generators can produce coherent present and future weather data series that could be used for predicting the agricultural, hydrological, ecological, and economic effects of the prospective climate change with the help of various impact models (e.g., crop models). 

The outputs of weather generators have been used many times in scientific studies: as inputs for crop models [1–5] and also in climate change studies [6–9]. The main hindrance of using and improving crop models is the lack of input data—including weather data—that is required for operating and testing these models. There are two situations when weather generator outputs could be especially useful for crop modellers: they can be applied for sites (1) where no meteorological data is available or (2) where only a short data series is available.

In the past decade, many meteorological stations were set up all over the world mainly owing to special (e.g., environmental modelling) projects, as well as to the decrease of purchase prices. Hence, there are plenty of automated weather stations that have only a couple of years long data series. A frequently applied presupposition concerning weather generators is that the minimum of 30 years, that is, the so-called climate normal period, is required for their adequate parameterization. The first objective of our study was to determine the minimum data series length required for parameterizing the MVWG [10] stochastic weather generator to produce sufficient quality weather data.

Certain applications (e.g., crop model aided decision support systems) require (at least) realistic weather data for locations with no measurements. These data could be produced by weather generators that were parameterized by using the measured data of the neighboring meteorological stations [11]. Thus, the second objective of our study was to investigate the conditions of obtaining sufficient quality weather data using the spatially interpolated results of the MVWG generator.

## 2. Materials and Methods

### 2.1. The MVWG Weather Generator

The recently developed multivariable stochastic weather generator, MVWG [10], was used in this study. Not repeating all the details [10], the main features of the MVWG are as follows: the daily precipitation amounts as well as the dry or wet status of a day are calculated independently of other meteorological variables. MVWG uses the “serial approach” [12] instead of Markov chain for generating consecutive dry and wet spells. 3-parameter Weibull function (1) is used for calculating the length of dry and wet spells. The same Weibull function (1) is introduced for approximating the distribution of daily precipitation amounts:

(1) CDF={1−e−(x−a/b)cx≥a0x<a,

where *a*, *b*, and *c* are parameters of the cumulative distribution function (CDF) that are to be determined by curve fitting.

Calculation of the rest of the daily variables (maximum and minimum temperature, global radiation, sunshine hours, relative humidity, cloudiness, wind speed, and atmospheric pressure) is conditioned on the dry or wet status of the day. The time series of each variable is reduced to a time series of residual elements by removing the annual course of means and standard deviations. The annual courses of means and standard deviations are approximated by a 3-order Fourier series. Residual series of the variables are calculated by using the weakly stationary generating process proposed by Matalas [13] where the auto- and cross-correlations of the generated variables are implemented by calculating lag-0 and lag-1 cross-correlation matrices monthly. The established monthly matrix equations are solved by applying the spectral decomposing theorem. A user friendly interface has been created for MVWG that enables the users to customize the parameterization procedure as well as handling the input and output data.

### 2.2. Data

Data of US meteorological stations from the *SAMSON* [14] database has been used in the study. Data series of each location were at least 20-year-long from the period of 1961 to 1990 and included daily global radiation, maximum and minimum temperature, and precipitation [15]. This database provides a very good basis for analyzing and comparing the performance of weather generators since it contains data of locations from different climates, and the covered period is free from explicit monotonous trends [16]. 

### 2.3. Minimum Data Series Length for Generator Parameterization

**Figure 1:**
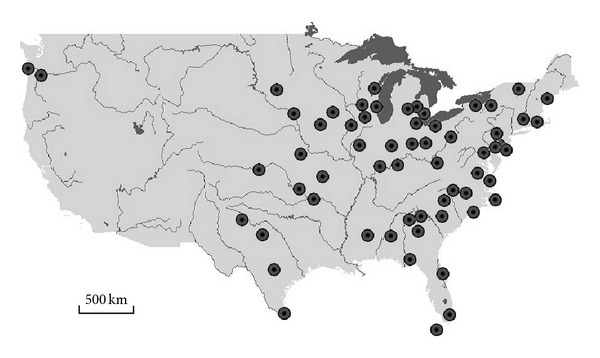
Location of the 61 meteorological stations which provided the data used in the study. These stations have complete data series for the period of 1961–1990.

A subset of 61 stations (Figure 1) having complete 30-year data series (1961–1990) has been collated from the *SAMSON* [14] database for finding the minimum data series length for generator calibration.

Eighty subseries were separated within the 30 year data series for each location (Figure 1): six 25-year-long series (1961–1985, 1962–1986,…, 1966–1990); eleven 20-year-long series (1961–1975, 1962–1976,…, 1971–1990); sixteen 15-year-long series (1961–1975, 1962–1976,…, 1976–1990); twenty-two 10-year-long series (1961–1970, 1962–1971,…, 1981–1990) and twenty six 5-year-long series (1961–1965, 1962–1966,…, 1986–1990). MVWG was parameterized using each subseries as well as the 30-year-long series, and 100-year-long synthetic data series were generated for every location using each parameterization. The generated series were compared to the observed 30-year-long series. The expected values of the monthly number of wet days, the cumulative solar radiation, average temperature, and cumulative precipitation values were compared for every location and for each month, which meant 61 × 12 = 732 comparisons for every climatic variable and for each parameterization. Mann-Whitney *U*-test [17] was used to determine whether the difference was significant or not in a comparison since the normality of the distributions could not be guaranteed. Zero, one, two or three were assigned to each comparison if the result of the *U*-test indicated nonsignificant (*P* ≥ 0.05), marginally significant (*P* < 0.05), significant (*P* < 0.01), or highly significant (*P* < 0.001) differences, respectively, between the observed and the synthetic data. The sum of the assigned values gave a total score (TS) for each climatic variable and for each parameterization, while the maximum score (MS) was 732 × 3 = 2196. An acceptability index (AI) was calculated for all climatic variables and for each parameterization using the following formula:

(2) $$\begin{array}{c} \text{AI}=100\cdot \left(1-\frac{\text{TS}}{\text{MS}}\right). \end{array}$$

The higher the acceptability index, the better the performance of the weather generator: AI = 100 means that no significant differences could be found between the observed and the generated data series of the climatic variable in question while AI = 0 means that the compared data series were significantly different for all the 61 sites and for every month. Acceptability indices for the corresponding (equally long) subseries were averaged. 

Then, the observed and the synthetic data series were used as crop model inputs. Corn yields and annual cumulative evapotranspiration values were calculated with the CERES-Maize crop model [18] for all of the investigated sites. Soil data of a loam profile and plant specific data of a FAO-400 cultivar were retrieved from the database of the DSSAT ver. 3.5 software package [19] and were given as inputs to the crop model. The simulation results obtained with the generated weather data were compared to those obtained with the observed data using *U*-test and the above defined acceptability index.

### 2.4. Spatial Interpolation of the Generator Results

**Figure 2:**
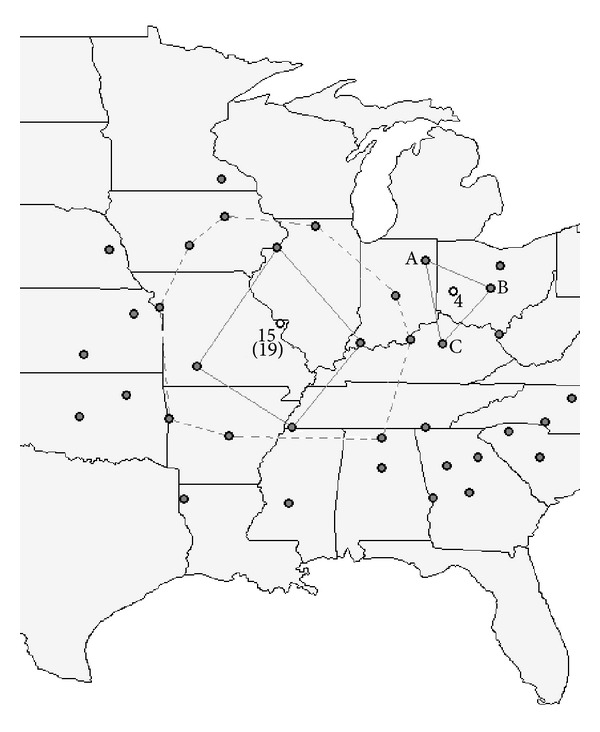
Location of the meteorological stations (4, 15, and 19 denote central points, and, A, B, and C denote border points) whose data used for testing the spatial interpolation method. Three example polygons are presented with central points 4, 15, and 19 (see Table 1). Polygon #15 and polygon #19 have the same central point but have 4 (solid line) and 9 (dashed line) border points, respectively.

Lam [20] and Burrough [21] have described a variety of quantitative interpolation methods suitable for computer algorithms which could be divided into exact (proximal, b-splines, kriging, etc.) and approximate (trend surface analysis, fourier series, distance weighed averaging, etc.) methods. A simple, point based approximate interpolation method with distance weighed average scheme (3) has been chosen for this study. From the available 38 weather stations (Figure 2), twenty polygons (Table 1) were formed in the Eastern and the central regions of US (three example polygons presented in Figure 2), defined by one central point and 3 to 9 border points. MVWG was parameterized for every border station, and the parameter values were interpolated into the central point using the following formula:

(3) $$\begin{array}{c} P^{C}=\sum_{i=1}^{N}\frac{d_{i}^{-1}}{\sum_{i=1}^{N}d_{i}^{-1}}\cdot P_{i}^{B}, \end{array}$$

where *P* ^*C*^: parameter for the central point, *P*_*i*_^*B*^: parameters for the *i*th border point, *d*_*i*_: distance between the central and the *i*th border point, and *N*: number of border points.

The result parameters of the interpolation were then used for generating 100-year-long data series for the central points of the polygons. The mean of the monthly values of the following climatic variables in the observed and the generated data series was compared: number of wet days; cumulative global radiation; average temperature; and cumulative precipitation. The synthetic and the observed data were compared by using *U*-test and the previously defined acceptability index with a maximum score of 3 × 12 = 36 since the comparisons were carried out on a monthly basis for each polygon.

The effect of the number of the border points (Figure 2, polygons 15 and 19 have the same central point having 4 and 9 border points, resp.), as well as of the average distance and altitude difference between the central and the border points (Table 1) on interpolation efficiency (measured by AI) was also investigated. The observed weather data, as well as the synthetic data series generated with the previously introduced interpolation technique were also used as crop model inputs for the central points of each polygon (Table 1). The rest of the model inputs were set according to Section 2.3. The simulated corn yield and evapotranspiration values, calculated by using the observed and generated weather data, were compared by using Student's *t*-tests.

## 3. Results and Discussion

### 3.1. Minimum Data Series Length for Generator Calibration

Dependence of the generator performance (acceptability indices) on the data series lengths that were used for parameterization is presented in Table 2. Though exact determination of the minimum required length is difficult, one can establish that in case of the generated meteorological variables even 15 years would provide fair approximations in over 95% of the cases (even considering significant and highly significant differences with double and triple weights, resp.). For crop model simulations, an at least 20-year-long observed data series is required for the same level of adequacy. The difference is likely caused by the nonlinear accumulation of the errors in the CERES crop model. One should also remark that 30 years provide a 100% exact simulation compared to the real observed series even in case of crop yield and evapotranspiration calculations. When the distributions of the AIs over the months or the sites were investigated no extraordinary month or station was found. The performance of the generator was practically the same for all of the months and stations. Thus, the results in Table 2 could be regarded to be quite robust, independent in the location and the period within the year.

Concerning the calculated yields, the nonsignificant, slightly significant and highly significant *U*-test results correspond with a 0–2%, 9–12% and 16–20% average relative errors, respectively. Crop modellers usually consider a 15% relative error in yield prediction to be an acceptable deviation. When 15-year-based artificial weather data were used for yield predictions, the proportion of the highly significant differences (average relative error greater than 15%) was only 2%. Based on these, one can be almost 100% certain that 15 years of observed weather data would be enough for adequately parameterizing the MVWG generator.

### 3.2. Spatial Interpolation of the Generator Results

The introduced spatial interpolation technique was not able to provide adequate parameters for generating realistic weather data for 5 polygons (Table 3). The failure of the interpolation can most likely be traced back to specific geographical features of the “problematic” polygons. The central point of the 3rd polygon, Waterloo, IA, USA (Table 1) is situated in the valley of the Cedar River. The southern spur of the Appalachian Mountains protrudes into the 6th polygon. The 7th and the 16th polygons cover large portions of the Boston Mountains and the Springfield Plateau, respectively. The parameters to be interpolated do not follow monotonous trends between the border points because of these geographic features which is a key assumption of the applied technique. Thus, the interpolation fails for these polygons. The 19th and the 20th have the same central point: St Louis, MO, USA. The size of these polygons may provide a plausible explanation for the weak performance of the interpolation. For example, the border points and the central point of the 20th polygon lay in different climatic zones within the USA: Lexington, KY, USA, and Shreveport, LA, USA, are in the Humid Subtropical region, Rochester and MN, USA, is in the Humid Continental (cool summer) region, while St Louis, MO, USA, is in the Humid Continental (warm summer) climatic region.

An interesting result can be found when the entries in Tables 3 and 4 are cross-checked. The interpolation technique performed worst for the 16th polygon (central point: Memphis, TN, USA) and was significantly worse than the next worst case: the 6th polygon (central point: Atlanta, GA, USA). Despite this, there were no significant differences in the crop model results at Memphis, while, in Atlanta, significantly different yield and evapotranspiration values were obtained with generated and with observed weather data. This apparent inconsistency can be resolved by the following arguments. In the applied crop model the relationship between the global radiation and the mass production is direct; the higher the radiation is, the more biomass is produced. The effect of the temperature, though it is more complex, is opposite. The higher the temperature is, the less biomass is produced due to increased heat stress, as well as increased water stress induced by elevated transpiration rates. Therefore, radiation and temperature take effect reversely. In Atlanta, the generated data underestimated global radiation and overestimated temperature, resulting in less mass production when the generated weather data was used for the simulations (Table 4). This is most likely caused by less available sunlight and increased evapotranspiration. In Memphis, the weather generator underestimated both the radiation and the temperature. Their effects counterbalanced each other; thus there was practically no difference (<0.3%) between the yield results obtained with observed and generated weather data. 

Note, that even for the worst case (polygon #20), the relative error of the yield estimations is smaller than 15%. For the 90% and 70% of the investigated polygons, the relative error is smaller than 10% and 5%, respectively.

For the rest of the investigated polygons (14 out of 20), the generator performance was quite convincing in estimating climatic parameters as well as yields. The investigated climatic parameters of the synthetic data series were statistically similar to those of the observed series. There were only two polygons with a more moderate performance with <90% accuracy for radiation. Despite this, no significant differences were found for these polygons when the crop model results were investigated (Table 4). 

No explicit relationship was found between the efficiency of the used interpolations technique and the investigated factors: the number of the border points, the average distance and altitude difference between the central and the border points. Despite this fact, the following rule of thumb could be observed. The efficiency of the interpolation (expressed in AI, Table 3) decreases as the average distance or the average altitude difference between the central and the border points increases (Table 1). One should note, however, that the above analysis has been performed for polygons with fairly even and low topography, where the network of existing stations may be representative enough. Regions with higher and more complex topography could behave differently especially with regard to the interpolation technique presented above.

## 4. Conclusions

This study was carried out to determine the minimum data series length required for adequate parameterization of the MVWG stochastic weather generator using the data of 61 meteorological stations in the USA. Based on a 15 year-long measured database, the MVWG is able to generate realistic weather data that can be used in meteorological as well as in modeling applications with high (95–100%) accuracy. Though this series length is most likely insufficient for accurately modeling the occurrence of extremes, it seems to be enough for mimicking the averages of the most important climatic parameters.

The applicability of a simple, point based approximate interpolation method with distance weighed average scheme to provide adequate weather generator parameters was also investigated. With the help of the presented interpolation technique, the MVWG could be used for generating realistic weather data for sites with no measurements situated in climatically and geographically homogeneous regions. The topography of the investigated midwest and southern regions of the US is generally less diverse than that of the western regions. The findings of this paper apply to somewhat homogenous terrains and should be tested for mountainous and very heterogeneous terrains as well.

## Figures and Tables

**Table 1:** Central points (CP) and border points (BP) of the polygons, defined for interpolation of the WG parameters, as well as the average distance (D_CP-BPs_) and the mean altitude difference (AD_CP-BPs_) between the central point and the border points.

#	Central point (CP)	Border points (BPs)	Number of BPs	D_CP-BPs_ (km)	AD_CP-BPs_ (m)
1	CHNC	COSC, GRNC, GRSC	3	132	88
2	COOH	DAOH, HUWV, MAOH	3	131	65
3	WAIA	DEIA, MOIL, ROMN	3	168	83
4	DAOH	COOH, FOIN, LEKY	3	157	37
5	LOKY	EVIN, ININ, LEKY, NATN	4	171	78
6	ATGA	AHGA, CHTN, COSC, MAGA	4	135	140
7	TUOK	FOAR, OKOK, SPMO, WIKS	4	202	160
8	COMO	DEIA, KAMO, MOIL, SPMO, STMO	5	247	75
9	HUAL	CHTN, BIAL, METN, NATN	4	182	34
10	TOKS	DEIA, OMNE, SPMO, WIKS	4	273	103
11	EVIN	ININ, NATN, STMO	3	235	81
12	LIAR	FOAR, JAMS, METN, SHLA, SPMO	5	267	79
13	DEIA	COMO, MOIL, ROMN, SIIA, TOKS	5	287	62
14	DEIA	MIMN, NOVA, SPIL, SPMO	4	389	104
15	STMO	EVIN, METN, MOIL, SPMO	4	321	91
16	METN	EVIN, HUAL, JAMS, LIAR, SPMO, STMO	6	335	90
17	FOTN	LIAR, OKOK, SHLA, SPMO, TUOK	5	244	138
18	EVIN	CHTN, HUAL, ININ, LEKY, METN, SPIL, STMO	7	312	90
19	STMO	DEIA, FOTN, HUAL, ININ, KAMO, LIAR, LOKY, ROIL, WAIA	9	442	72
20	STMO	LEKY, ROMN, SHLA	3	625	151

Abbreviations for the stations: AHGA: Athens, GA; ATGA: Atlanta, GA; BIAL: Birmingham, AL; CHNC: Charlotte, NC; CHTN: Chattanooga, TN; COOH: Columbus, OH; COMO: Columbia, MO; COSC: Columbia, SC; DAOH: Dayton, OH; DEIA: Des Moines, IA; EVIN: Evansville, IN; FOAR: Fort Smith, AR; FOIN: Fort Wayne, IN; FOTN: Fort Smith, TN; GRNC: Greensboro, NC; GRSC: Greenville, SC; HUAL: Huntsville, AL; HUWV: Huntington, WV; ININ: Indianapolis, IN; JAMS: Jackson, MS; KAMO: Kansas City, MO; LEKY: Lexington, KY; LIAR: Little Rock, AR; LOKY: Louisville, KY; MAGA: Macon Lewis, GA; MAOH: Mansfield, OH; METN: Memphis, TN; MIMN: Minneapolis, MN; MOIL: Moline, IL; NATN: Nashville, TN; NOVA: Norfolk, VA; OKOK: Oklahoma City, OK; OMNE: Omaha, NE; ROIL: Rockford, IL; ROMN: Rochester, MN; SHLA: Shreveport, LA; SIIA: Sioux City, IA; SPIL: Springfield, IL; SPMO: Springfield, MO; STMO: St Louis, MO; TOKS: Topeka, KS; TUOK: Tulsa, OK; WAIA: Waterloo, IA; WIKS: Wichita, KS.

**Table 2:** Acceptability indices for the investigated monthly climatic variables as well as for the investigated crop model results.

Series length, year	5	10	15	20	25	30
WD	77.8	91.5	96.1	98.2	99.0	99.3
CRad	68.9	89.0	96.2	99.0	99.8	100.0
AvT	69.5	90.2	96.9	99.4	99.9	100.0
CPrec	74.1	91.2	97.1	98.8	99.5	99.7
CET	68.5	81.4	92.6	95.0	98.5	100.0
Yield	60.8	87.0	91.5	94.2	100.0	100.0

WD: number of wet days, CRad: cumulative solar radiation, AvT: average temperature, CPrec: cumulative precipitation, CET: annual cumulative evapotranspiration.

**Table 3:** Acceptability indices obtained when the observed weather series were compared to the generated series for central points of the investigated polygons. The parameters for generating artificial weather data were obtained by spatial interpolation using the data of the border points.

#	AI_CPobs-CPgen_		
	Global Radiation	Average Temperature	Precipitation
1	100	100	97.2
2	94.4	100	100
3	97.2	83.3	100
4	94.4	97.2	100
5	100	91.7	100
6	86.1	44.4	94.4
7	97.2	41.6	97.2
8	80.5	94.4	100
9	97.2	100	100
10	97.2	91.7	100
11	97.2	97.2	100
12	97.2	97.2	100
13	97.2	94.4	100
14	100	100	100
15	100	100	100
16	61.1	22.2	91.7
17	94.4	100	100
18	86.1	100	100
19	100	72.2	100
20	88.9	61.1	100

**Table 4:** Differences in the crop model results obtained by using observed and generated weather data of the central points of the investigated polygons. The parameters for generating artificial weather data were obtained by spatial interpolation using the data of the border points. Asterisk denote significant differences (significance level, *α* = 0.05).

#	Yield difference, kgha^−1^		Relative yield difference, %	Evapotranspiration, mmy^−1^
1	307		3.79	22
2	5		0.05	4
3	−1073*		8.22	−12
4	194		1.86	11
5	159		1.82	−4
6	−724*		8.94	26
7	494		7.00	24
8	592		5.64	27
9	−43		0.45	13
10	−207		2.12	−3
11	133		1.54	10
12	−214		2.85	−3
13	−125		1.24	−10
14	−137		1.36	−10
15	−7		0.07	6
16	20		0.24	5
17	121		1.51	10
18	138		1.60	13
19	1143*		11.91	17
20	1356*		14.13	21
